# Antimicrobial peptides from *Capsicum chinense* fruits: agronomic alternatives against phytopathogenic fungi

**DOI:** 10.1042/BSR20200950

**Published:** 2020-08-21

**Authors:** Layrana de Azevedo dos Santos, Gabriel Bonan Taveira, Marciele Souza da Silva, Rodrigo da Silva Gebara, Lídia da Silva Pereira, Jonas Perales, André Teixeira-Ferreira, Érica de Oliveira Mello, André de Oliveira Carvalho, Rosana Rodrigues, Valdirene Moreira Gomes

**Affiliations:** 1 Laboratório de Fisiologia e Bioquímica de Microrganismos, Centro de Biociências e Biotecnologia, Universidade Estadual do Norte Fluminense Darcy Ribeiro, Campos dos Goytacazes, RJ, Brazil; 2 Laboratório de Toxinologia, Fundação Osvaldo Cruz (FIOCRUZ), Rio de Janeiro, RJ, Brazil; 3 Laboratório de Melhoramento e Genética Vegetal, Centro de Ciências e Tecnologias Agropecuárias, Universidade Estadual do Norte Fluminense Darcy Ribeiro, Campos dos Goytacazes, RJ, Brazil

**Keywords:** Defensin, Fusarium, lipid transfer protein, mechanism of action, Plant peptides

## Abstract

In recent years, the antimicrobial activity of peptides isolated from a wide variety of organs from plant species has been reported. However, a few studies have investigated the potential of antimicrobial peptides (AMPs) found in fruits, especially *Capsicum chinense* (pepper). The present study aimed to purify and characterize peptides from *Capsicum chinense* fruits and evaluate their inhibitory activities against different phytopathogenic fungi and also analyze the possible mechanisms of action involved in microbial inhibition. After fruit protein extraction and high-performance liquid chromatography (HPLC), different fractions were obtained, named F1 to F10. Peptides in the F4 and F5 fractions were sequenced and revealed similarity with the plant antimicrobial peptides like non-specific lipid transfer proteins and defensin-like peptide. The F4 and F5 fractions presented strong antimicrobial activity against the fungus *Fusarium solani* and *Fusarium oxysporum*, causing toxic effects on these fungi, leading to membrane permeabilization, endogenous reactive oxygen species increase, activation of metacaspase and loss of mitochondrial function.

## Introduction

Pepper (*Capsicum* spp.) represents one of the most important vegetables in the world due to its high versatility with wide range of applications in pharmaceutical, cosmetic, decoration, and culinary industries [1]. The genus *Capsicum* belongs to the Solanaceae family, commonly known as pepper or paprika; this genus has a wide genetic diversity, composed of 38 described species, including 22 semidomesticated or wild species and only five domesticated species (*C. annuum* var. *annuum* L., *C. baccatum* var. *pendulum, C. chinense* Jacq., *C. frutescens* L. and *C. pubescens* Ruiz & Pav.) [2].

Although the genus *Capsicum* is considered an important component of the fresh vegetable market, its production is limited in the face of numerous fungal, bacterial, and viral infections that affect its cultivation, causing severe quantitative and qualitative losses [3]. The strategies used to control these pathogenic microorganisms involve the continuous and indiscriminate use of chemical fungicides, leading to residues that not only are toxic to pepper plants but also pollute the environment. Moreover, the unrestrained use of these fungicides has become increasingly ineffective due to the resistance development against many of these compounds by pathogens [4]. There are currently serious concerns about the increasing resistance of pathogens and the use of pesticides and the negative impacts that they are causing to human health and environment [5]. In this context, AMPs (antimicrobial peptides) appear to be a promising alternative to overcome this problem. AMPs are small molecules produced by all living organisms and have gained considerable attention due to their potent antimicrobial activity against a broad range of microbes. Moreover, some kill microorganisms rapidly at low concentrations [6]. Plant AMPs share some common characteristics, including a low molecular mass (<10 kDa), amphipathic properties, a net positive charge at physiological pH and the presence of cysteine residues interconnected in pairs, forming disulfide bonds that confer high stability to these molecules. Due to the amphipathic characteristic of many AMPs, they have the ability to interact electrostatically with biological membranes. Regarding the mechanism of action, AMPs can act in membrane permeabilization, altering its porosity, and may consequently act on intracellular targets [7]. Plant AMPs are divided into different families according to their structural characteristics namely cyclotides, protease inhibitors, thionins, lipid transfer proteins (LTPs) and plant defensins [8]. LTPs are small peptides that comprise two families. The LTPs that form the more studied family, the family one, have a primary structure of 90 to 95 amino acid residues and are basic, presenting isoeletric points (pI) of between 9 and 10 [9]. Plant defensins have a primary structure of 45 to 54 amino acid residues, with a low molecular weight of approximately 6 kDa. Defensin also contain several basic amino acids endowing the molecule with a positive charge at a neutral pH [10].

Recent studies have shown that some pepper species have peptides with strong antimicrobial activity against bacteria [11], yeasts [12], filamentous fungi [13] and have inhibitory activity against α-amylases [14] and proteases [15]. The J1-1/GST defensin of *Capsicum* showed strong inhibitory activity against fungi of the genus *Colletotrichum*, which cause anthracnose, an infectious disease that despite the efforts to control it, remains an endemic disease, resulting in large reductions in annual yields worldwide [16]. In 2017, Taveira et al. [17] demonstrated that a thionin isolated from *Capsicum annuum* fruits, called *Ca*Thi, has a strong antifungal effect against *F. solani*, a saprophytic fungus that causes numerous diseases in plants. *Ca*Thi was able to cause plasma membrane permeabilization, oxidative stress, activation of caspases and loss of viability in *F. solani*. Additionally, those authors showed that the combined treatment of *Ca*Thi and fluconazole can be a strong candidate for studies to improve the control of *F. solani*. The aforementioned examples demonstrate the importance of peppers as a source of AMPs, which can have agricultural applications, such as control of diseases caused by microorganisms. In this work, we purified and isolated peptides from *C. chinense* fruits, evaluated their inhibitory effects on the phytopathogenic fungi and analyzed their possible mechanism of action.

## Materials and methods

### Fruit obtention

*Capsicum chinense* Jacq., (accession UENF 1755) seeds were provided by the Laboratório de Melhoramento Genético Vegetal (LMGV), at the Centro de Ciências e Tecnologias Agropecuárias (CCTA), Universidade Estadual do Norte Fluminense Darcy Ribeiro (UENF), Campos dos Goytacazes, Rio de Janeiro, Brazil. For the planting, the seeds were sown in a Styrofoam tray of 72 cells containing commercial substrate (4% nitrogen; 14% phosphorus; 8% potassium) (Vivatto, Brazil) and maintained in a growth chamber with a controlled temperature of 28°C and a photoperiod of 12 h light/dark. After the emergence of two pairs of definitive leaves, seedlings were transplanted to 5 l pots and transferred to a greenhouse. The plants were irrigated once a day until mature fruits be collected.

### Microorganisms

*Fusarium oxysporum* (5845), *Fusarium solani* (4014), *Colletotrichum lindemuthianum* (5771) and *Colletotrichum gloeosporioides* (5522) phytopathogenic fungi were maintained and cultured in Sabouraud agar (10 g/l peptone, 2 g/l glucose, 20 g/l agar) (Merck Millipore Brazil) in the LFBM, at the Centro de Biociências e Biotecnologia (CBB), UENF, Campos dos Goytacazes, Rio de Janeiro, Brazil.

### Peptides from *C. chinense* fruits – extraction and fractionation

Protein extraction of *C. chinense* fruits and peptides fractionation were carried out according to the method described by Taveira et al. [12] with modifications. Briefly, 80 g of *C. chinense* fruits (without seeds) was extracted for 2 h (at 4°C) in 400 ml of extraction buffer (10 mM NaH_2_PO_4_; 15 mM Na_2_HPO_4_; 100 mM KCl; 1.5% pH 5.4) (Sigma) and then precipitated between 0 and 70% relative ammonium sulfate (Merck). The precipitate was resuspended in distilled water and heated at 80°C for 15 min. The resulting suspension was clarified by centrifugation (10,000 × ***g*** for 15 min at 4°C), and the supernatant was extensively dialyzed against distilled water and lyophilized. This material obtained was called F/0-70 fraction and subjected to fractionation by HPLC system (Prominence, Shimadzu) using a C18 reversed-phase column (Shim-pack VP-ODS, Shimadzu) attached to a C8 pre-column (Pelliguard, Sigma-Aldrich). The column was equilibrated and run at a flow rate of 0.5 ml.min^−1^ with solvent A (aqueous 0.1% TFA) (Sigma-Aldrich) for the first 2 min, then 0–10% solvent B (100% propanol (Merck Millipore Brazil) in 0.1% TFA) for 8 min. For 94 min, the concentration of solvent B increased from 10% to 50% and was maintained at that concentration for 2 min, and finally, the concentration of solvent B was reduced to 0% and maintained at this concentration until the end of the run at 110 min. Proteins elution were monitored by online measurement of the absorbance at 220 nm, using a photodiode array detector (SPD-M20A, Shimadzu). The obtained fractions were collected in 2 ml microcentrifuge tubes (Eppendorf, Germany) and named F1, F2, F3, F4, F5, F6, F6, F7, F8, F9, and F10 (with respective retention times of 21′83″, 24′63″, 33′13″, 36′13″, 39′24″, 54′20″, 63′75″, 66′39″, 71′07″, and 73′32″).

### SDS-tricine-gel electrophoresis

SDS-tricine-gel electrophoresis was performed as previously described by Schägger and Von Jagow [18]. For visualization of proteins, gels were stained with silver nitrate, according to the methodology described by Morrissey [19]. Ultra-low ranger molecular weight marker (Sigma-Aldrich) was used as protein molecular weight marker.

### Preparation of fungal inoculum and growth inhibition assay

For the preparation of conidia from *F. oxysporum, F. solani, C. lindemuthianum* and *C. gloeosporioides*, fungal cultures were grown on a Petri dish containing Sabouraud 2% glucose agar (Merck Millipore Brazil) for 11 days at 30°C. After this period, 10 ml of Sabouraud broth (10 g/l peptone, 2 g/l glucose) (Merck) was added to the dishes, and the conidia were gently released with the aid of a Drigalski spatula. This suspension was filtered to eliminate mycelial debris. Conidia were quantified in a Neubauer chamber (Laboroptik, United Kingdon) under an optical microscope (Axioplan Imager.A2, Zeiss). Subsequently, fungal conidia (2000 cells.ml^−1^) were incubated in 200 µl of Sabouraud broth containing different peptide fractions from *C. chinense* fruits at 100 and 200 µg.ml^−1^. The assay was performed in 96-well microplates (NUNC, Nunclon Surface) at 30°C. Optical readings were taken at 620 nm (EZ Read 400 Research/Biochrom). The assay lasted up to 24 or 30 h, depending on the growth of the fungal species tested. The entire assay was performed in triplicate under aseptic conditions in a laminar flow hood according to the methodology described by Broekaert et al. [20] with modifications.

### Amino acid sequence analysis

For internal sequencing of the peptides present in the F4 and F5 fractions, which showed the highest antimicrobial activity among the tested microorganisms, the peptides, they were separated by SDS-tricine-gel electrophoresis, the protein bands of interest were extracted and digest from the gel and subjected to a mass spectrometry evaluation [21]. In brief, the peptides digest by trypsin was first co-crystalized with a large molar excess of the α-cyano-4-hydroxycinnamic acid matrix before being analyzed by matrix-assisted laser desorption time-of-flight mass spectrometry (MALDI-TOF-MS). The instrument used was an AB SCIEX TOF/TOF^™^ 5800 System spectrometer (AB SCIEX) in the reflection mode. Sequences were compared in amino acid databases and submitted to auto-aligned using the BLAST program [22].

### Effect of fractions on plasma membrane permeabilization

The permeabilization of the plasma membrane of fungi treated with F4 and F5 fractions (fractions with the highest antimicrobial activity) isolated from *C. chinense* fruits was measured by Sytox green (Molecular Probes Invitrogen, Life Technologies) uptake, as described by Thevissen et al. [23] with some modifications. This particular dye can only penetrate a cell when its membrane is compromised; it has a high affinity for nucleic acids and emits green fluorescence when bound to them. After 24 h of the growth inhibition assay, 100 µl of fungal cells suspension grown in the presence and in the absence of F4 and F5 fractions (200 µg.ml^−1^) were incubated with 0.2 µM Sytox green. After a 30 min incubation at 30°C with constant agitation at 500 rpm, the cells were observed under an optical microscope (Axioplan Imager.A2, Zeiss) coupled to an AxioCam MRc5 (Zeiss) camera, and the images were analyzed by the Axiovision software version 4.0 (Zeiss). The microscope was equipped with a set of fluorescent filters for fluorescein detection (excitation wavelength between 450 and 490 nm and emission of 500 nm). All fluorescence images were obtained with the same exposure time.

### Reactive oxygen species detection assay

To evaluate whether the mechanism of action of F4 and F5 fractions involves the induction of reactive oxygen species (ROS), we used the fluorescent probe 2´,7´-dichlorofluorescein diacetate (H_2_DCFDA) (Calbiochem - EMD). This dye enters the cell passively, is deacetylated by intracellular esterases and, being oxidized by ROS, becomes fluorescent. Thus, after 24 h of the growth inhibition assay, aliquot (100 µl of the suspension of fungal cells) was incubated with 20 μM of the H_2_DCFDA probe (Calbiochem), for 2 h at 30°C with constant agitation at 500 rpm, as described by Mello et al. [24] After this period, the cells were observed and analyzed under an optical microscope, as described in “Effect of fractions on plasma membrane permeabilization”.

### Caspase activity detection assay

Detection of caspase activity was performed using the CaspACE FITC-VAD-FMK In Situ Marker (Promega), as described by the manufacturer. After 24 h of the inhibition assay, fungal cells (grown in the presence and in the absence of 200 µg.ml^−1^ of F4 and F5 fractions) were resuspended, washed once in 500 μl of PBS (10 mM NaH_2_PO_4_, 0.15 M NaCl) pH 7.4 and resuspended in 50 μl of staining solution (supplied by the kit) containing 50 μM marker. After incubation for 20 min at 30°C with constant agitation at 500 rpm, the cells were washed in 500 μl PBS and resuspended in 20 μl PBS. Then, the cells were observed and analyzed under an microscope, as described in ‘Effect of fractions on plasma membrane permeabilization’.

### Analysis of mitochondrial functionality

Mitochondrial functionality was assessed by the fluorescent dye Rhodamine 123 (Sigma-Aldrich). After 24 h of the growth inhibition assay, fungal cells grown in the presence and in the absence of F4 and F5 fractions (200 µg.ml^−1^) were resuspended, incubated with 10 μg.ml^−1^ of Rhodamine 123 with constant agitation at 500 rpm for 2 h at 30°C, protected from light and then analyzed by differential interference contrast (DIC) under an optical microscope equipped with a fluorescence filter (excitation wavelength of 506 nm, emission wavelength of 530 nm). All fluorescence images were obtained with the same exposure time.

### Statistical analysis

All data were analyzed using one-way ANOVA. Mean differences at *P*<0.05 were considered significant. All statistical analyses were performed using GraphPad Prism software (version 5.0 for Windows).

## Results

### Extraction and fractionation of fruit antimicrobial peptides

**Figure 1:**
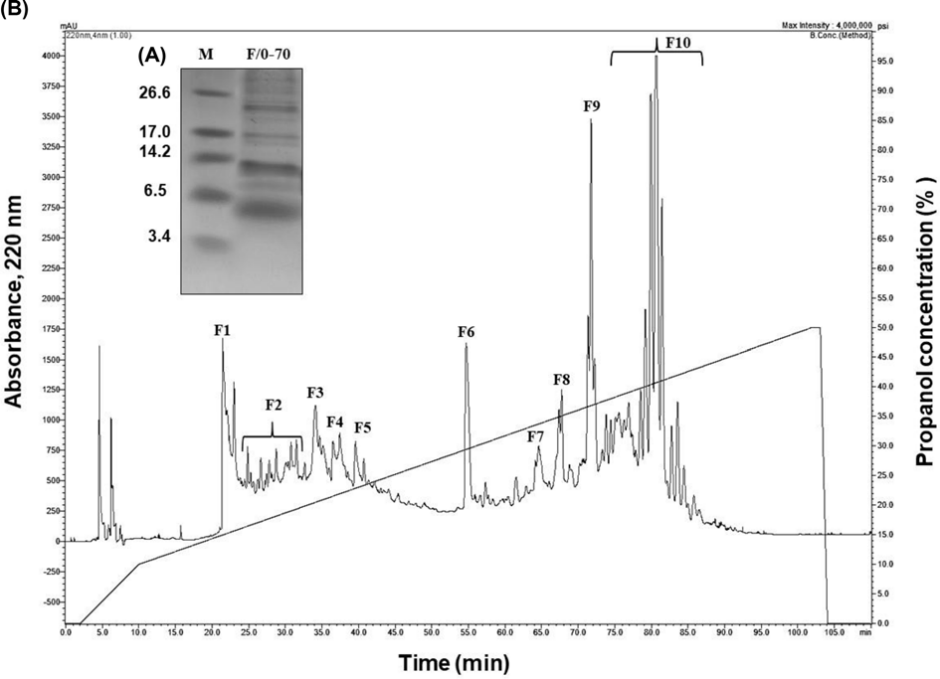
Purification of antimicrobial peptides from *Capsicum chinense* (**A**) Chromatogram of the F/0-70 fraction from *C. chinense* fruits in C18 reversed-phase column. The column was equilibrated and run with 0.1% TFA (Solvent A) and eluted using a linear gradient of 100% propanol in 0.1% TFA (Solvent B), flow 0.5 ml.min^−1^. (**B**) Electrophoretic visualization in a SDS-Tricine-gel of F/0-70 fraction from *C. chinense* fruits treated with β-mercaptoethanol; *M*, Molecular mass marker (kDa).

**Figure 2:**
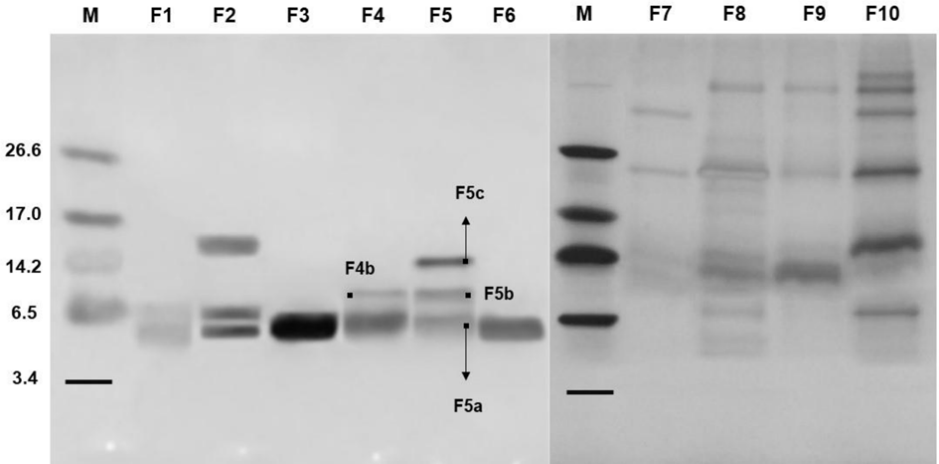
Tricine gel electrophoresis from *C. chinense* after reversed-phase chromatography Electrophoretic visualization in a SDS-Tricine-gel of peptides enriched fractions obtained after fractionation of F/0-70 from *C. chinense* fruits by C18 reversed-phase chromatography in HPLC system. All fractions were treated with β-mercaptoethanol. The proteins bands were stained with silver nitrate; *M*, Molecular mass marker (kDa). F4b, F5b and F5c – isoforms of lipid transfer proteins. F5a – defensin-like peptide.

Electrophoretic visualization of the F/0-70 fraction proteins showed the presence of low-molecular-mass bands (Figure 1A). The F/0-70 fraction was separated by HPLC system and yielded a chromatogram with ten major fractions denominated as F1, F2, F3, F4, F5, F6, F7, F8, F9 and F10 (Figure 1B), predominantly composed of peptides with molecular masses between 5 and 14 kDa (Figure 2). F1 and F4 fractions showed a profile with two peptides each, with molecular masses of approximately 5 and 6 kDa. F2 and F5 fractions presented three major peptides, one with a molecular mass of approximately 5 kDa and two with a molecular mass between 6 and 14 kDa. F3 and F6 fractions are composed of one unique peptide with molecular masses of approximately 5 kDa. F7, F8, F9 and F10 fractions presented a profile with protein bands of molecular mass between 3 and over 26 kDa, as determined by SDS-tricine-gel electrophoresis.

### Effect of fractions on the growth of phytopathogenic fungi

**Figure 3:**
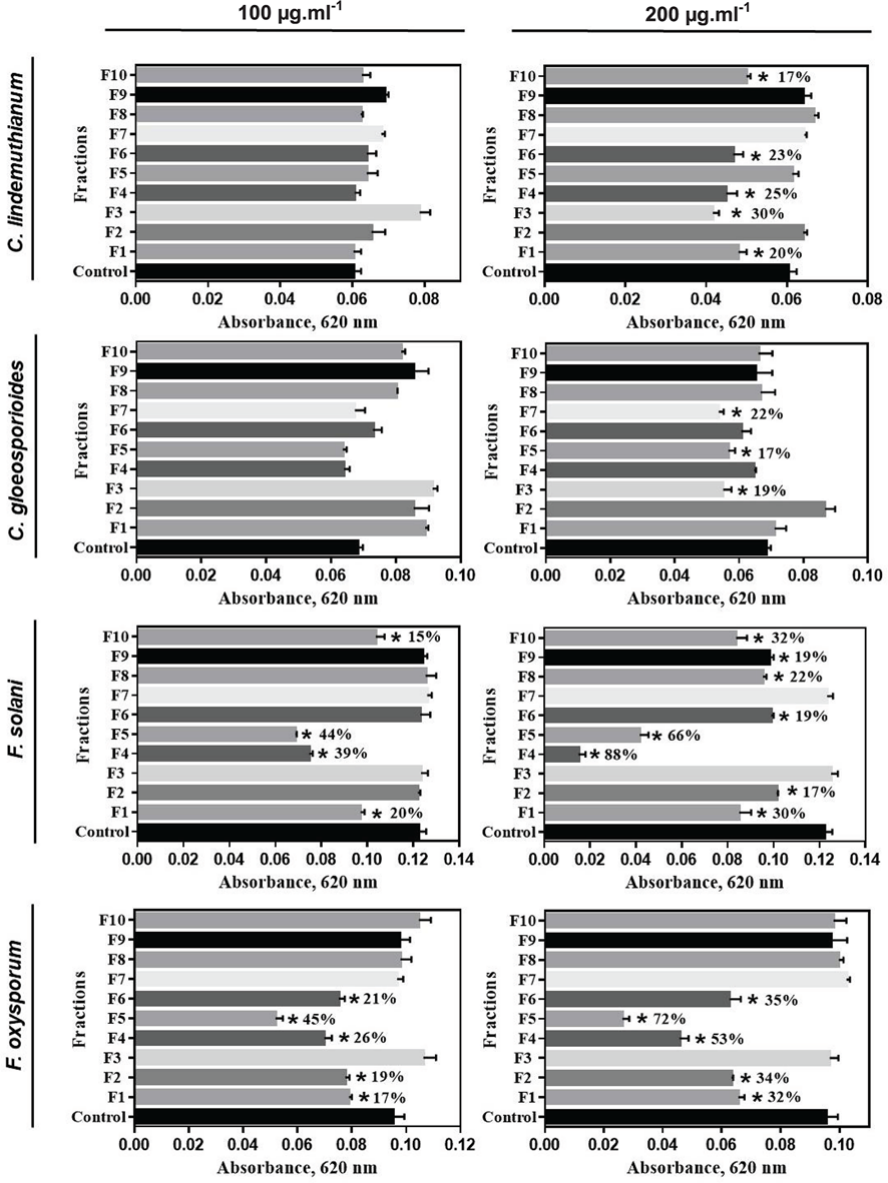
Effect of the *C. chinense* fractions on filamentous fungi growth Growth inhibition assay of different filamentous fungi in the presence of 100 and 200 μg.ml^−1^ of fractions rich in peptides obtained after C18 reversed-phase column. The assay was observed until 24 or 30 h. The values are the means (and ±SD) of triplicates. Asterisks indicate significant differences (*P*<0.05) between the experimental treatment and the control.

The different fractions obtained after C18 reversed-phase column were tested against the fungi *C. lindemuthianum, C. gloeosporioides, F. oxysporum* and *F. solani* (Figure 3). As shown in Figure 3, no inhibition of *C. lindemuthianum* growth was observed for any of the fractions tested at the concentration of 100 μg.ml^−1^. However, when we tested the fractions at 200 μg.ml^−1^, the F1, F3, F4, F6 and F10 fractions caused significant reductions (*P*<0.05) of 20, 30, 25, 23 and 17%, respectively, in the growth of this filamentous fungus. We also analyzed the inhibitory effect of all fractions against *C. gloeosporioides*, at the concentration of 100 μg.ml^−1^ none of the fractions tested were able to inhibit fungal growth. However, at 200 μg.ml^−1^, F3, F5 and F7 fractions caused growth inhibition (*P*<0.05) of 19, 17 and 22%, respectively. For *F. solani*, a significant inhibitory effect of 20, 39, 44 and 15% was observed in the presence of 100 μg.ml^−1^ of the F1, F4, F5 and F10 fractions, respectively. At the concentration of 200 μg.ml^−1^, significant inhibition of 30, 17, 88, 66, 19, 22, 19 and 32% (*P*<0.05) was observed for F1, F2, F4, F5, F6, F8, F9 and F10 fractions, respectively. When we analyzed the inhibitory effect of all ten fractions at a concentration of 100 μg.ml^−1^ against *F. oxysporum*, we observed that F1, F2, F4, F5 and F6 fractions were able to cause significant growth reduction (*P*<0.05) of 17, 19, 26, 45 and 21%, respectively. Moreover, at 200 μg.ml^−1^, these same fractions were able to significantly inhibit (*P*<0.05) fungal growth, with 31, 34, 53, 72 and 35% inhibition, respectively. The other fractions (F3, F7, F8, F9 and F10) did not significantly inhibit the growth of this fungus at any of the concentrations tested.

Thus, we could observe that F4 and F5 fractions were the most toxic against fungi of the genus *Fusarium*, at concentration of 200 μg.ml^−1^.

### Amino acid sequence analysis of peptide fractions by mass spectrometry

**Figure 4:**
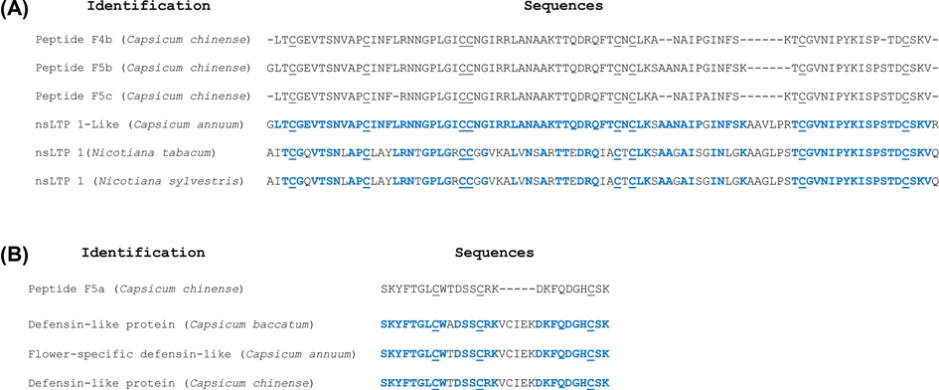
Alignment of amino acid residues from *C. chinense* fractions Alignment of the amino acids residues of peptides with other similar peptides described. The sequences were obtained from SWISS-PROT and aligned by Clustal Omega. (**A**) The F4b (*Cc*-LTP4b), F5b (*Cc*-LTP5b) and F5c peptides (*Cc*-LTP5c) showed similarity with the sequences: Non-specific lipid-transfer protein 1-like *Capsicum annuum* (NCBI Reference Sequence: XP 016559796.1); Nonspecific lipid-transfer protein 1 *Nicotiana tabacum* (NCBI Reference Sequence: pdb 1T12 A Chain A); Non-specific lipid-transfer protein 1 *Nicotiana sylvestris* (NCBI Reference Sequence: XP 009761744.1). (**B**) The F5a peptide (*Cc*-Def5a) showed similarity with the sequences: Defensin-like protein *Capsicum baccatum* (NCBI Reference Sequence: PHT42128.1); Flower-specific defensin-like *Capsicum annuum* (NCBI Reference Sequence: XP 016579688.1); Defensin-like protein *Capsicum chinense* (NCBI Reference Sequence: PHU10972.1). Identical residues are shown in blue (Cys residues included). The conserved Cys residues were underlined and gaps (-) were included to improve alignment.

Due to the high antimicrobial activity presented by the F4 and F5 fractions, the peptides contained in these fractions (indicated in Figure 2) were subjected to amino acid sequencing by mass spectrometry (Figure 4). Sequence analysis from peptides F4b, F5b and F5c revealed similarity with non-specific lipid transfer proteins (nsLTP). In Figure 4A we observed that the analysis of the F4b peptide resulted in a sequence fragment with 80 amino acid residues and the alignment showed 80, 63 and 52% similarity with non-specific lipid-transfer protein 1-like from *C. annuum*, nonspecific lipid-transfer protein 1 from *Nicotiana tabacum* and non-specific lipid-transfer protein 1 from *Nicotiana sylvestris*, respectively. For this reason, F4b has been renamed to *Cc-*LTP4b. The F5b peptide was obtained a sequence of 84 amino acid residues with 84, 66 and 55% similarity to non-specific lipid-transfer protein 1-like from *C. annuum*, nonspecific lipid-transfer protein 1 from *N. tabacum* and non-specific lipid-transfer protein 1 from *N. sylvestris*, respectively. We renamed F5b to *Cc-*LTP5b. Already for F5c peptide was obtained 80 amino acid residues with 79, 62 and 51% similarity also with a non-specific lipid-transfer protein 1-like from *C. annuum*, nonspecific lipid-transfer protein from 1 *N. tabacum* and non-specific lipid-transfer protein 1 from *N. sylvestris*, respectively. The F5c peptide was designated *Cc*-LTP5c. By performing the alignment of the sequences we saw that peptides F4b and F5b have 95% similarity between each other, peptides F5b and F5c present 94% and peptides F4b and F5c present 96% similarity between their sequences, and present eight cysteine residues (Cys) conserved.

For F5a peptide was also selected and compared with protein databases, and it showed 24, 25 and 24% identity with the primary structure to defensin-like protein from *C. baccatum*, flower-specific defensin-like from *C. annuum* and defensin-like protein from *C. chinense*, respectively. We renamed F5a peptide to *Cc*-Def5a. The sequence alignments also revealed that three cysteine residues are conserved (Figure 4B).

### Effect of fractions on plasma membrane permeabilization

**Figure 5:**
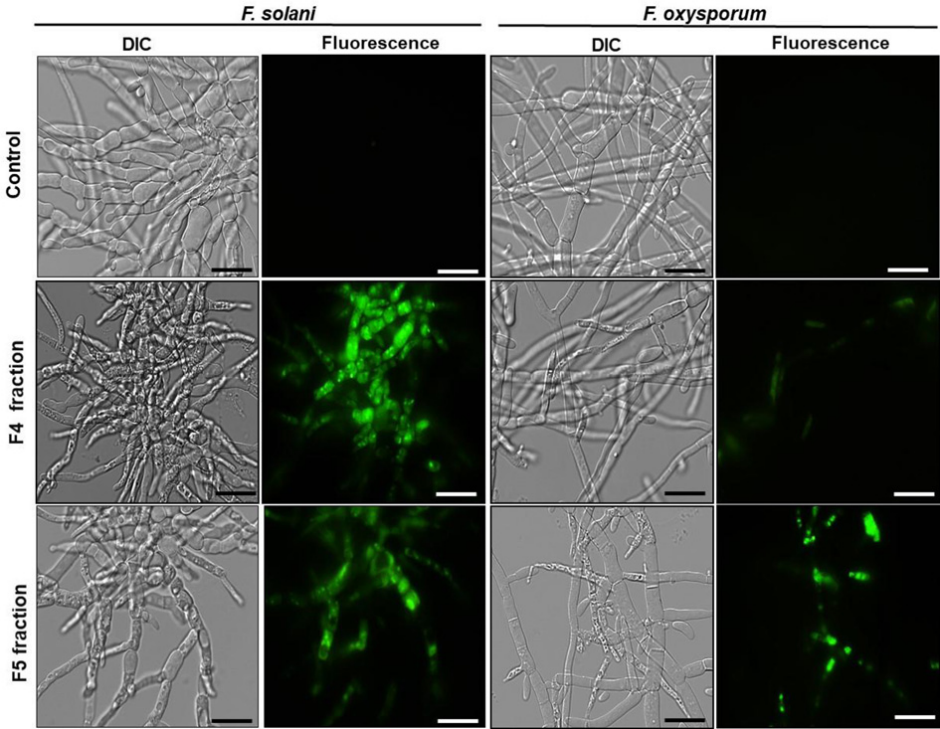
Membrane permeabilization assay Assay performed with fluorescence microscopy of cells of different filamentous fungi treated with the fluorescent probe Sytox green to evaluate membrane permeabilization. Control cells (grown in the absence of fractions) and treated cells with F4 and F5 fractions for 24 h. The assay were performed at the concentration of 200 µg.ml^−1^ and the cells were visualized by DIC and fluorescence; bars = 50 μm.

Based on the results obtained, we evaluated the ability of F4 and F5 fractions to promote the permeabilization of the membranes of *F. solani* and *F. oxysporum* fungi using Sytox green dye. After 24 h, the fungi grown in the absence (control) and presence of the fractions (200 μg.ml^−1^) were incubated with the fluorescent dye Sytox green. Figure 5 shows fluorescence for the two fungi when cultured in the presence of F4 and F5 fractions. In contrast, the control did not show any fluorescence, suggesting that these fractions acted on the plasma membrane of these fungi, damaging its structure and allowing dye staining, thus indicating membrane permeabilization. In general, in the clear field images (DIC) in Figure 5, we also observe differentiated growth of the fungi treated with the fractions, with morphological changes as follows: more branched or shorter hyphae with elongation deficiencies and a reduction in the number of hyphae, which were not observed in the controls.

### Reactive oxygen species detection assay

**Figure 6:**
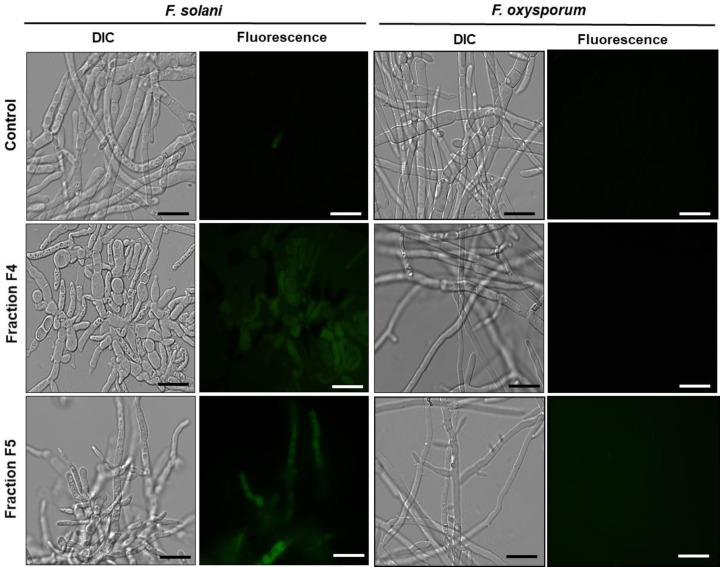
Reactive oxygen species (ROS) detection assay Oxidative stress assay of cells of different filamentous fungi incubated with 2′,7′-dichlorofluorescein diacetate (H_2_DCFDA) for 2 h to evaluate the increase in the ROS production. Control cells (grown in the absence of fractions) and treated cells with F4 and F5 fractions for 24 h. The assay were performed at the concentration of 200 µg.ml^−1^ and the cells were visualized by DIC and fluorescence; bars = 50 μm.

After the growth inhibition assay, *F. solani* and *F. oxysporum* hyphae were incubated with the H_2_DCFDA probe for the detection of endogenous ROS production. As shown in Figure 6, we observed dye labeling in cells grown in the presence of F4 and F5 fractions (200 μg.ml^−1^), indicating that these fractions increased ROS production in *F. solani*. For *F. oxysporum*, it was shown that F4 and F5 fractions (200 μg.ml^−1^) were not able to cause an increase in ROS production. In controls grown in the absence of fractions, no fluorescence was observed.

### Caspase activity detection assay

**Figure 7:**
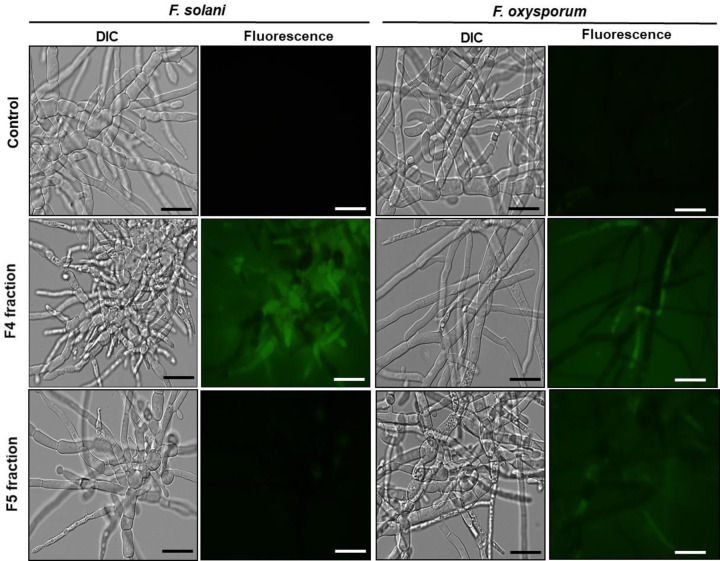
Caspase activity detection Images of detection of caspase activity assay of cells of different filamentous fungi after being incubated with CaspACE FITC-VAD-FMK probe. Control cells (grown in the absence of fractions) and treated cells with F4 and F5 fractions for 24 h. The assays were performed at the concentration of 200 µg.ml^−1^ and the cells were visualized by DIC and fluorescence microscopy; bars = 50 μm.

To analyze whether the fractions induced apoptotic events, the activity of caspase-type enzymes was assessed. After a 24 h inhibition assay, the fungal hyphae of *F. solani* and *F. oxysporum* grown in the absence (control) and in the presence of the F4 and F5 fractions (200 μg.ml^−1^) were incubated with the CaspACE FITC-VAD-FMK *in situ* marker (Figure 7). For the fungus *F. solani*, treatment with fraction F4 resulted in the activation of caspases as indicated by the strong fluorescent labeling of these cells. However, when treated with the F5 fraction, caspase activity was not detected and the control did not show fluorescence as well. Additionally, the fungus *F. oxysporum* treated with either F4 or F5 showed weak fluorescence, indicating the induction of caspase, unlike the controls that did not show any fluorescence.

### Analysis of mitochondrial functionality

**Figure 8:**
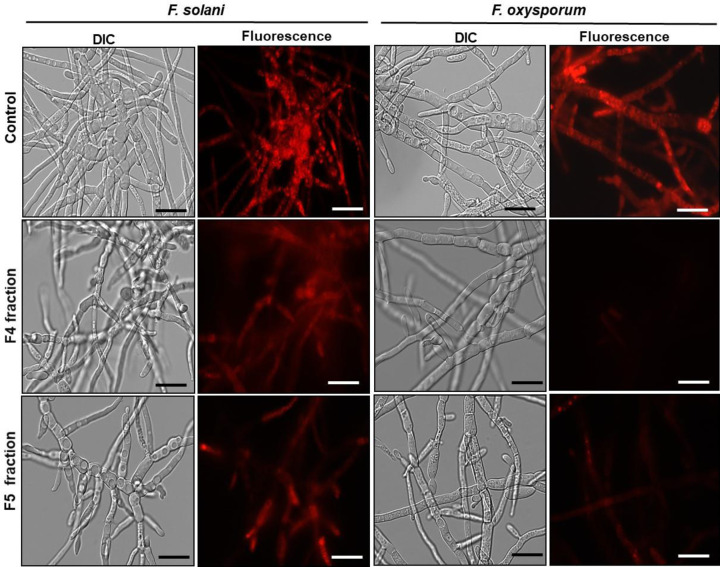
Mitochondrial functionality assay Images of mitochondrial functionality assay of cells of different filamentous fungi after being incubated with Rhodamine 123 probe. Control cells (grown in the absence of fractions) and treated cells with F4 and F5 fractions for 24 h. The assay were performed at the concentration of 200 µg.ml^−1^ and the cells were visualized by DIC and fluorescence microscopy; bars = 50 μm.

In this work, we also performed a mitochondrial functionality assay using the fluorescent dye Rhodamine 123 (Figure 8). After a 24 h inhibition assay, the fungus *F. solani* treated with 200 μg.ml^−1^ of F4 and F5 fractions presented functional mitochondria, observed by the strong fluorescence of the dye Rhodamine 123, as also shown for the control cells (grown in the absence of the fraction). However for *F. oxysporum* we observed that both fractions (F4 and F5) (200 μg.ml^−1^) caused decrease mitochondrial activity, as indicated by the weaker red fluorescent staining in comparison to the control.

## Discussion

Currently, threats to plant health caused by pathogenic microorganisms constantly limit agricultural production [25]. With the aim of improving plant production, the use of compounds produced naturally by plants appears to be a sustainable and promising alternative against these plant pathogens (e.g., viruses, bacteria and fungi), which have developed resistance against many bactericides and fungicides used to control plant diseases [26]. In this context, because the importance of the medicinal properties and mainly our interest in the antimicrobial activities already shown in the literature for *Capsicum* sp, in this work, we isolated and characterized AMPs of *C. chinense* fruits and evaluated their mechanism of action on microorganisms of agronomic interest. Initially, due to the presence of peptides in the F/0-70 fraction of *C. chinense* (Figure 1A) and antifungal activity (data not shown). This fraction was separated by C18 reversed-phase column (Figure 1B) and the ten fractions obtained (Figure 2), presented antifungal activity against four different species of phytopathogenic fungi (Figure 3). In recent years, our group has reported several plant AMPs as potent growth inhibitors of microorganisms [27–29]. Games et al. [27] purified a defensin from *Phaseolus vulgaris* seeds, called *Pv*D1, which showed inhibitory activity against yeasts and phytopathogenic fungi at concentration of 100 µg.ml^−1^. Taveira et al. [12] observed, at different concentrations, the potent activity of a peptide isolated from *C. annuum* fruits (named *Ca*Thi) against the yeasts *S. cerevisiae, C. albicans* and *C. tropicalis* and the bacteria *Escherichia coli* and *Pseudomonas aeruginosa*. Based on these and several other studies carried out by us and different research groups [14,29–31], in addition a limited quantity of the peptide fractions we chose to use only two higher concentration concentrations (100 and 200 µg.ml^−1^) of peptides fractions from *C. chinense* against filamentous fungi. Herein, we verified that fractions presented a higher inhibitory activity on fungal growth when tested at the concentration of 200 µg.ml^−1^ and more efficiently inhibited fungi of the genus *Fusarium* than those of the genus *Colletotrichum*, especially F4 and F5 fractions. Santos et al. [13] isolated the Fa5 fraction of *C. annuum* fruits, which showed high antimicrobial activity only against species of the genus *Fusarium*. In contrast, Silva et al. [28] showed that *CaTI*, a proteinase inhibitor isolated from *C. annuum* seeds, was able to inhibit only the growth of fungi of the genus *Colletotrichum*. Slazak et al. [32] isolated several cytotoxins from *Viola odorata*, namely, cyO2, cyO3, cyO13 and cyO19, and showed that all these showed inhibitory activity against all fungi tested (*F. oxysporum, F. graminearum, F. culmorum, Mycosphaerella fragariae*, and *Botrytis cinerea*). These results show that in general, AMPs can inhibit some pathogens but not others, which will depend on not only the concentration used but also the peptide and fungal species tested [33]. Moreover, the capacity of synergistic interactions between plant peptides present in different fractions on pathogen inhibition has been reported in the literature; such synergistic interactions are frequent and of great value for the antimicrobial action of AMPs [10]. Carvalho et al. [34] showed synergistic interactions between LTP and defensin, both isolated from cowpea seeds, which in combination strongly inhibited fungal growth *F. oxysporum* and *F. solani*. Chen et al. [35] reported that tomato transformed with the β-1,3-glucanase and defensin genes presented increased resistance to *Ralstonia solanacearum*. Fardin et al. [36] evaluated the synergy between two fractions obtained from *Bertholletia excelsa* seeds and observed that the isolated fractions eliminated 27.9% (H3 fraction) and 27.7% (H4 fraction) of *Leishmania amazonensis* cells, but in synergism, the fractions eliminated 97.7% of these cells. Through database analysis was showed that the peptides identified from fractions F4 and F5 were nsLTP (*Cc*-LTP4b, *Cc*-LTP5b and *Cc*-LTP5c) with high sequence similarity to each other (eluted at different retention times), and a defensin-like (*Cc*-Def5a) with degree of similarity to other peptides isolated from *Capsicum* (Figure 4). Diz et al. [37] and Maracahipes et al. [29] reported the amino acid sequence of plant LTP and defensin, respectively, by mass spectrometry sequencing method. In those two articles, shown a LTP and defensin present in *C. annuum* and their antimicrobial activities against fungi. Bard et al. [14] isolated two similar vicillins-like peptides present in a single fraction of *C. baccatum*, that exhibited strong antifungal activity against several yeasts. Taveira et al. [12] isolated two thionins-like peptides of two fractions from *C. annuum* fruits with identity of 42% with each other.

Considering the results obtained, we began to investigate the possible mechanism of action of F4 and F5 fractions, responsible for the inhibition of *F. solani* and *F. oxysporum* growth. The exact mechanism of action exerted by AMPs is not yet fully understood, but it is believed that most AMPs inhibit or kill microorganisms through membrane interactions [9,38]. We demonstrated that F4 and F5 fractions structurally compromised the membranes of the fungi *F. solani* and *F. oxysporum*, causing their permeabilization (Figure 5). A peptide belonging to the thionin family, viscotoxin A3 (VtA3), has also been shown to be capable of altering the permeability of the plasma membrane, and it has also been observed that this peptide is internalized by *F. solani* cells [39]. Many works report that after membrane interaction, AMPs can trigger a series of subsequent events such as the induction of reactive oxygen species (ROS), the inhibition of protein synthesis, the inhibition of mitochondrial activity and the triggering signaling cascades leading to apoptosis [40,41]. Taveira et al. [42] showed *Ca*Thi has strong antimicrobial activity against six *Candida* species and caused plasma membrane permeabilization in all tested yeasts. In addition, *Ca*Thi was also reported to be located intracellularly in nuclei, and to induce an increase in intracellular ROS production in *C. tropicalis*.

ROS are considered primary regulators of cell death and are linked to many crucial apoptotic pathways in yeasts. Studies have shown that an increase of ROS in the medium may be toxic to organisms, leading to the destruction of various cell types by means of apoptosis [43,44]. In this work, increased ROS production was observed only in *F. solani*, suggesting that increased oxidative stress induced by F4 and F5 fractions may underlie the growth inhibitory effect on this fungus. Nevertheless, oxidative stresses were not detected for *F. oxysporum* implicating that we could not associate the fractions role and ROS production with growth inhibition of this fungus, under the conditions studied (Figure 6). Some authors show that increased production of ROS in target organisms is a recurring mechanisms of action employed by AMPs [39,24]. However, cell death may be an independent event, that occur individually or complementary to other mechanisms of action [45], as observed in the present study. Vieira et al. [40] showed that *Lp*-Def1 defensin, isolated from *Lecythis pisonis* seeds, inhibited *C. albicans* growth, caused membrane permeabilization and mitochondrial functionality loss in this yeast. However, no endogenous increase in ROS was observed. In view of this information, our next objective was to verify if apoptotic events could be taking place when the *F. solani* and *F. oxysporum* were treated with the peptide fractions. Apoptosis is a type of programmed cell death, characterized by noticeable biochemical and physical alterations that occur in the cytoplasm and in cell components, which is regulated by a complex network of proteins and metabolic pathways. The central core of this process is regulated by a family of proteins named caspases [46]. Caspases are specific cysteine-containing aspartate proteases, and are typically activated in the early stages of apoptosis [47]. In these two assays, it was possible to observe that only the F4 fraction activated caspases (Figure 7) but did not cause dissolution of the mitochondrial membrane potential in *F. solani* (Figure 8). For *F. oxysporum*, it was observed that both fractions (F4 and F5) activated caspases (Figure 7), and caused dissipation of the mitochondrial membrane potential in this fungus (Figure 8), suggesting that the attack mechanism involved by these fractions presents different pathways against the studied fungi. An *HsAFP1* a defensin from *Heuchera sanguinea* was also able to induce intracellular accumulation of ROS, and activated caspases were required to cause apoptosis in *C. albicans* cells [48]. A synthetic plant defensin (γ_33_-_41_*Pv*D_1_^++^) was also able to activate metacaspases in *C. buinensis* cells and yet cause a collapse of the mitochondrial membrane potential in these cells [41]. Taveira et al [49]. showed that *Ca*Thi also triggered events that led to the death of the yeast *C. tropicalis*, such as increased endogenous ROS production, the presence of active caspases, the dissipation of the mitochondrial membrane potential and regulation of the external pH of the yeast cells. It is important to note that, probably, the peptides do not interact directly with the metacaspases, but these are activated probably due to the endogenous increase in ROS caused by the action of the peptides as described by Soares et al. [44] that demonstrated that the inhibition of the generation of ROS inhibits the death of the tested fungi to which was involved metacaspase activation.

In conclusion, in the present study, peptide fractions were obtained from *C. chinense* fruits. We show the capacity of these *Capsicum* fractions in inhibiting the growth of phytopathogenic fungi, which indicate an important role in the plant defense. In the present study, we also show that *C. chinense* fruits contains defensin-like peptide and isoforms of lipid transfer proteins which inhibit the growth and cause permeabilization of the membrane, induction of endogenous ROS, activation of caspases and functional collapse of mitochondria of genus *Fusarium*. Thus, this work reveals the great potential present in *Capsicum* fruits in defense response, aiming to contribute to the planning of new antifungal drugs.

## Abbreviations

AMP: antimicrobial peptide
HPLC: high-performance liquid chromatography
LTP: lipid transfer protein
ROS: reactive oxygen species
